# Supplementary figures and images for: Transcriptional alterations of peanut root during interaction with growth-promoting Tsukamurella tyrosinosolvens strain P9
Source: PLoS One. 2024 Feb 15;19(2):e0298303. doi: 10.1371/journal.pone.0298303 (PMC10868839; doi:10.1371/journal.pone.0298303)

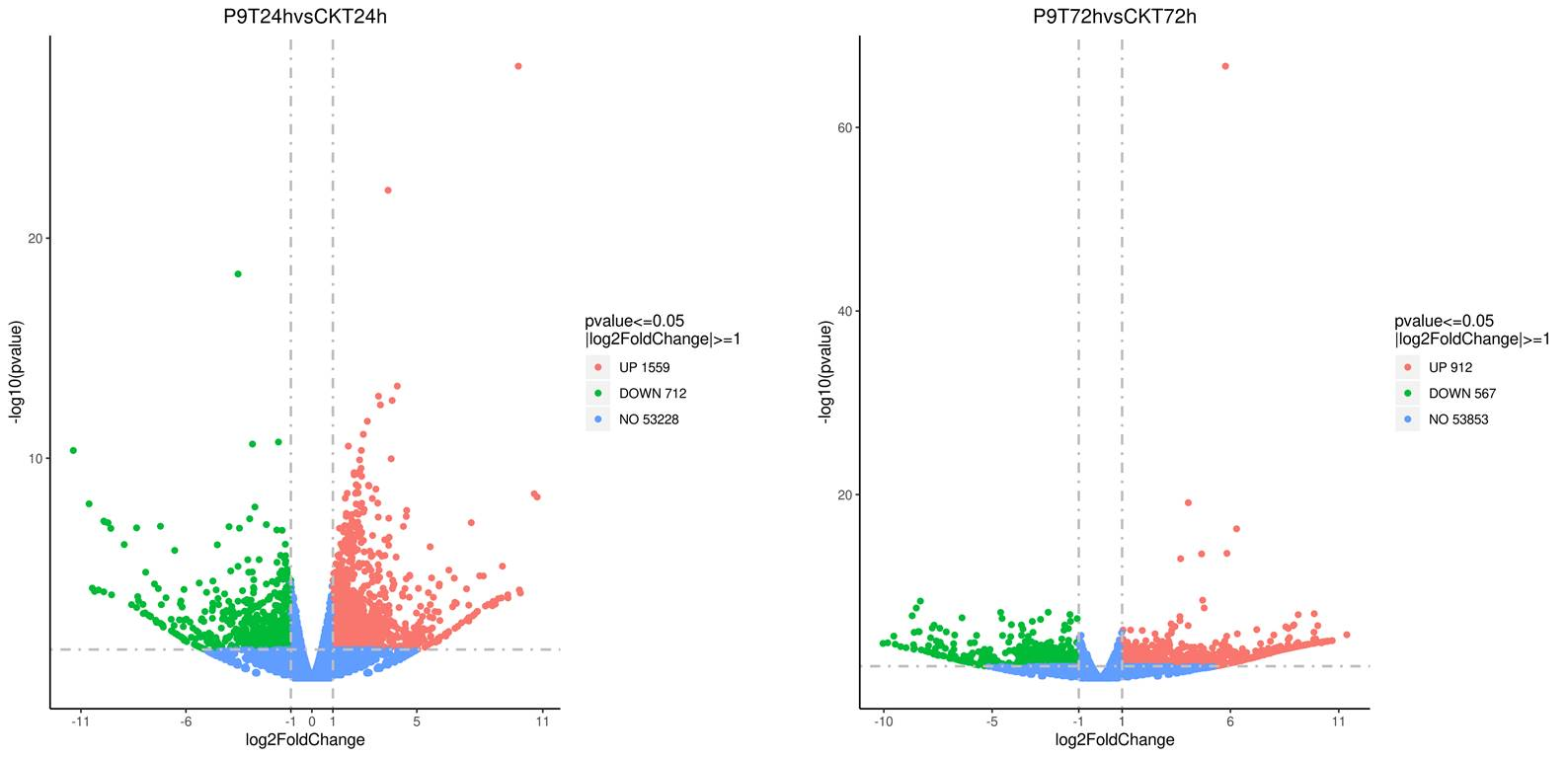

Supplement: S1 Fig — Plots of differential gene volcanoes in peanut roots inoculated with Tsukamurella tyrosinosolvens P9 for 24 h (left) or 72 h (right). Green and red dots indicate down-regulated and up-regulated differentially expressed genes (DEGs), respectively, and blue dots represent genes with no significant expression differences. (TIF) [file pone.0298303.s001.tif]

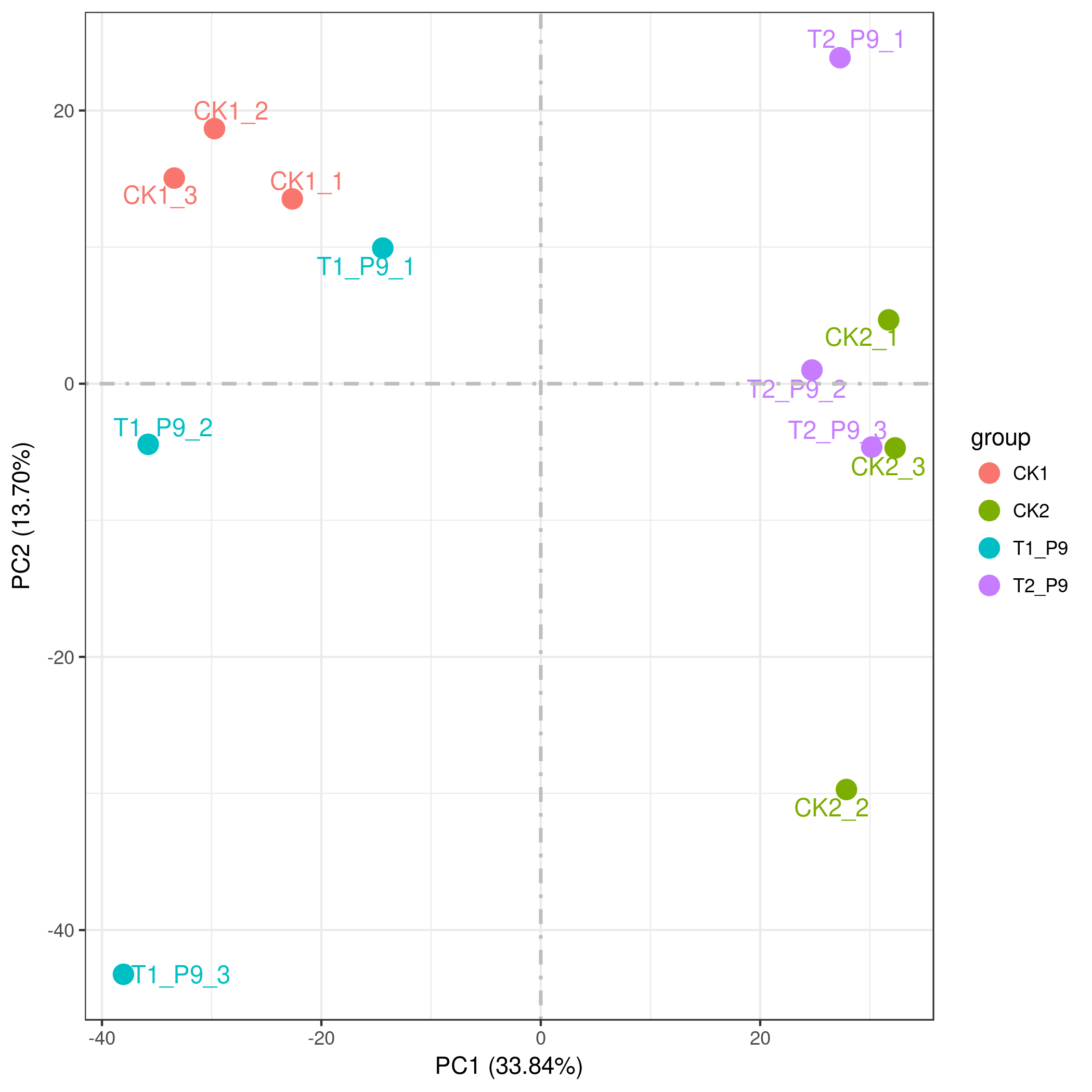

Supplement: S2 Fig — CK1 and CK2 are control groups at 24 and 72 h, respectively, and T1-P9 and T2-P9 are peanut groups inoculated with P9 for 24 and 72 h, respectively. Each treatment includes three replicates. (TIF) [file pone.0298303.s002.tif]
