# Supplementary material for: Transcriptional alterations of peanut root during interaction with growth-promoting Tsukamurella tyrosinosolvens strain P9
Source: PLoS One. 2024 Feb 15;19(2):e0298303. doi: 10.1371/journal.pone.0298303 (PMC10868839; doi:10.1371/journal.pone.0298303)
Supplement: Attachment — Submitted filename: Review Report_PONE-D-23-21487.docx [file pone.0298303.s010.docx]

The study titled "Transcriptome Alterations of Peanut Revealing the Growth-Promoting Molecular Mechanisms of *Tsukamurella tyrosinosolvens* P9," conducted by Lizhen Han and and co-workers established a a co-culture system between *Tsukamurella tyrosinosolvens* strain-P9 and peanut plants. They performed a transcriptome analysis of peanut roots interacting with strain P9 at two different time points. The primary aim was to investigate how peanut seedlings respond at the molecular level to the influence of strain P9 and to provide direct evidence supporting an in-depth analysis of the growth-promoting mechanisms employed by strain P9 on peanuts.

Their previous study reported *Tsukamurella tyrosinosolvens* P9 exhibits strong phosphate solubilization, indole-3-acetic acid (IAA) secretion, and siderophore production. Which transcripts are directly related to phosphate solubilization and siderophore production? The growth-promoting molecular mechanism of strain P9 is not clear in the present investigation, which which should be addressed in future revisions.

Why the authors chose the genome of *Arachis hypogaea* (assembly arahy.Tifrunner.gnm1.KYV3; GenBank accession number GCA_003086295.2) as a reference guided identification of transcriptome from the RNA-seq data. Silihong, an early maturing, local peanut variety with a dark red seed coat was used as the plant material in the present investigation. Based on the classification of cultivated peanuts, the “Silihong” variety belongs to which group (Spanish bunch type or Verginia runner type), that may be mentioned. Given the published and publicly available genome sequences of these subgroups of the *Arachis* species [Zhuang, Weijian, et al. Nature genetics 51, no. 5 (2019): 865-876; Bertioli, David J. et al. Nature genetics 51, no. 5 (2019): 877-884; Chen, Xiaoping, Molecular plant 12, no. 7 (2019): 920-934] selecting the appropriate reference genome is crucial for accurate transcriptome analysis.

The authors should explicitly define the rationale for selecting only 12 genes for qRT-PCR validation except for the GO (Gene Ontology) and KEGG (Kyoto Encyclopedia of Genes and Genomes) analyses. Moreover, qRT-PCR validation is an important aspect of any transcriptomics study. The data needs to be incorporated in the main text instead of supplementary data. If possible the raw qRT PCR experimental data may be shown in the supplementary file. Additionally, the authors should clarify whether there are any known connections between these 12 genes in the growth-promoting molecular mechanisms of the *Tsukamurella tyrosinosolvens* P9 strain. A comprehensive analysis of the transcriptome data except for the discussion of log_2_ fold change data is lacking in the present investigation.

While the authors claim that the DEG (differentially expressed gene) and KEGG (Kyoto Encyclopedia of Genes and Genomes) enrichment analyses revealed spatiotemporal differences in gene expression in peanut roots in response to strain P9, they have not provided experimental evidence to support this claim. Furthermore, the qRT-PCR validation was conducted only on root transcripts, and the study lacks validation in other tissues to confirm the spatiotemporal distribution of the identified transcripts from the RNA-seq data.

The raw RNA-seq data need to be submitted to the data repository and should also be available.

Lastly, there are some minor corrections needed in the manuscript, such as using "indices" instead of "indexes" in line numbers 141 and 162, italicizing the scientific name "Tsukamurella tyrosinosolvens" consistently throughout the manuscript, and italicizing gene names. Additionally, "Ca2+" should be written correctly in the whole manuscript.
