# Supplementary material for: Transcriptional alterations of peanut root during interaction with growth-promoting Tsukamurella tyrosinosolvens strain P9
Source: PLoS One. 2024 Feb 15;19(2):e0298303. doi: 10.1371/journal.pone.0298303 (PMC10868839; doi:10.1371/journal.pone.0298303)
Supplement: Attachment — Submitted filename: Review Report_PONE.docx [file pone.0298303.s012.docx]

The manuscript has undergone revisions, incorporating a modified title, “Transcriptional alterations of peanut root during interaction with growth-promoting *Tsukamurella tyrosinosolvens* strain P9”. Numerous comments have been addressed except a few.

The primary objective of the study was to explore the molecular response mechanism of peanut seedlings under the influence of strain P9 and to provide direct evidence for an in-depth analysis of the growth-promoting mechanism of strain P9 on peanuts. Their previous study reported that *Tsukamurella tyrosinosolvens* P9 exhibits strong phosphate solubilization, indole-3-acetic acid (IAA) secretion, and siderophore production. However, the corresponding genes were not extensively discussed except for the role of auxin in the revised manuscript. The root characteristics have a positive correlation with phosphate utilization and the phosphate solubilization also depends on the production of organic acids that helps in maintaining the pH of the environment for phosphate utilization. The modified version of the manuscript could be enhanced by addressing these issues, aligning with the authors' goal of elucidating the growth-promoting molecular mechanism of strain P9.

In response to this concern, the authors acknowledge that they did not delve deeply into the genes associated with the growth-promoting molecular mechanisms of *Tsukamurella tyrosinosolvens* P9. They appreciate the suggestion and express openness to further studying the growth-promoting molecular mechanisms of the P9 strain. However, the query arises about the significance of the present investigation if the molecular mechanism is not thoroughly addressed, considering the title "Transcriptional alterations of peanut root during interaction with growth-promoting *Tsukamurella tyrosinosolvens* strain P9."

Moreover, on page 9, line 165, there is a question about the term "multiple metabolisc pathways," with a suggestion that it might be a typographical error, possibly intended to be "metabolic."
