# Supplementary material for: Transcriptional alterations of peanut root during interaction with growth-promoting Tsukamurella tyrosinosolvens strain P9
Source: PLoS One. 2024 Feb 15;19(2):e0298303. doi: 10.1371/journal.pone.0298303 (PMC10868839; doi:10.1371/journal.pone.0298303)
Supplement: Attachment — Submitted filename: Response to Reviewers.doc [file pone.0298303.s013.doc]

Dear Editor,

Please find attached a revised version of our manuscript “Transcriptional alterations of peanut root during interaction with growth-promoting *Tsukamurella tyrosinosolvens* strain P9”.

Thank you for your kind letter regarding our manuscript, and the referees’ remarks. We have revised and modified the MS in accordance with the every reviewers’ comments, and revisions in the text are shown using yellow highlight for additions, and strikethrough font for deletions. And we also responded point-by-point to reviewer comments as listed below. Accordingly, we changed the serial number of the Reference because of some reference’s addition.

We hope that the revisions in the manuscript and our accompanying responses will be sufficient to make our manuscript suitable for publication in PLOS ONE. We shall look forward to hearing from you at your earliest convenience.

Yours sincerely,

First author, Xue Bai;

Corresponding author, Lizhen Han.

Corresponding author E-mail: [lzhan1@gzu.edu.cn;](mailto:lzhan1@gzu.edu.cn;) [hanlizhen11@163.com](mailto:hanlizhen11@163.com)

**Responses to the comments of Reviewer 1**

I appreciate the effort the authors have put into incorporating the suggested changes. It's evident that the authors have taken the feedback seriously, and the manuscript is progressing well. However, I would like to draw your attention to sentence construction and grammatical errors.

1. There are still some grammatical errors and spelling mistakes throughout the manuscript (for example: Line 58, 182, 185, 192, 188, 205, 250, 332, 333, 364, 367, 377, 420, 466, 499, 505, 549, 558, 561, 634, 651, 656, 658, 665). The use of the phrase, “in regard to” has been used excessively, making the story monotonous. I suggest using a grammar checking software to resolve this.

***Response:***

*Thanks for your suggestion. We are sorry about for that grammatical errors and spelling mistakes. We examined carefully and modified all these sections. And we changed “regarding”, “about”, “as for ” instead of “in regard to”, too..*

2. Line 65: Please correct the spelling of peanut type. It should be“Virginia runner”.

***Response:***

*Thanks, "Verginia runner type" has been changed to "Virginia runner type" in Line 61 of the revised manuscript.*

3. The link given in data availability statement is not working as of 27.11.23. Overall, I believe the manuscript is moving in the right direction, and I appreciate your dedication to refining the content..

***Response:***

*I am sorry, an open access time was set when the database was uploaded, now the database has been opened with the link of* [*https://www.ncbi.nlm.nih.gov/bioproject/PRJNA991079.*](https://www.ncbi.nlm.nih.gov/bioproject/PRJNA991079.) *And we modified this linkage in Line110.*

**Responses to the comments of Reviewer 2**

The manuscript has undergone revisions, incorporating a modified title, “Transcriptional alterations of peanut root during interaction with growth-promoting Tsukamurella tyrosinosolvens strain P9”. Numerous comments have been addressed except a few.
The primary objective of the study was to explore the molecular response mechanism of peanut seedlings under the influence of strain P9 and to provide direct evidence for an in-depth analysis of the growth-promoting mechanism of strain P9 on peanuts. Their previous study reported that Tsukamurella tyrosinosolvens P9 exhibits strong phosphate solubilization, indole-3-acetic acid (IAA) secretion, and siderophore production. However, the corresponding genes were not extensively discussed except for the role of auxin in the revised manuscript. The root characteristics have a positive correlation with phosphate utilization and the phosphate solubilization also depends on the production of organic acids that helps in maintaining the pH of the environment for phosphate utilization. The modified version of the manuscript could be enhanced by addressing these issues, aligning with the authors' goal of elucidating the growth-promoting molecular mechanism of strain P9.
In response to this concern, the authors acknowledge that they did not delve deeply into the genes associated with the growth-promoting molecular mechanisms of Tsukamurella tyrosinosolvens P9. They appreciate the suggestion and express openness to further studying the growth-promoting molecular mechanisms of the P9 strain. However, the query arises about the significance of the present investigation if the molecular mechanism is not thoroughly addressed, considering the title "Transcriptional alterations of peanut root during interaction with growth promoting Tsukamurella tyrosinosolvens strain P9."

***Response:***

*Thanks for your suggestion. We added the paragraph in Line578-603 to associated changes in the transcriptome of peanut root with the growth-promoting properties of P9 in phosphorus solubilizing and siderophore secretion. And accordingly, we added cited articles in References. That is, “Our previous study confirmed that the strain P9 has a good ability to dissolve phosphorus and produce siderophore.* *Phosphorus is an essential element for the growth and development of plants. In order for plants to use insoluble phosphate in the environment, it must first be dissolved through the action of organic acids [Brito et al. 2020]. During the early stage of co-culture with strain P9, the PDC and ALDH genes, which catalyse the production of pyruvate and acetic acid, respectively, were up-regulated. Additionally, the LPIN, PDAT, phospholipase C and lysophospholipase II genes, which are involved in glycerophospholipid and glycerolipid metabolism, were also up-regulated. In the later stage, the genes encoding PDC and ADH was up-regulated to a greater extent (7.92-fold and 8.35-fold, respectively), and the genes encoding D-lactate dehydrogenase, malate dehydrogenase and pyruvate decarboxyase, which catalyse the production of lactic acid and malic acid, and the genes encoding choline-phosphate cytase and GPAT, which are involved in the synthesis of phospholipids and phosphoinositides, were significantly up-regulated. Our additional study also confirmed that organic acids are one of the main components of root extracts from peanut seedlings inoculated with the P9 strain [Jiang et al. 2023]. So, it was speculated that the P9 strain not only promoted the formation of organic acids in peanut roots, but also provided more soluble phosphorus for peanuts through its phosphorus solubilsing ability, which obviously promoted plant root development. In addition, iron is closely related to chlorophyll synthesis and photosynthesis, and the regulation of iron homeostasis greatly affects cell signal transduction, which in turn affects root growth [Kobayashi et al. 2019; Liang 2022], but the efficiency of iron utilization by plants is very low. Siderophore secreted by PGPR are an effective strategy for plants to absorb and utilize iron [Tsai and Schmidt 2017]. After inoculation with the P9 strain, the chlorophyll content of the leaves increased, root development was more vigorous, and the expression of photosynthesis-related proteins PetC and PetF was up-regulated. It was speculated that the siderophore secreted by P9 provided sufficient iron for the growth of peanut seedlings, promoting the development of peanut roots and an increase in chlorophyll content. This was consistent with the good plant growth-promoting ability of the P9 strain”.*

Brito LF, López MG, Straube L, Passaglia LMP, Wendisch VF. Inorganic phosphate solubilization by rhizosphere bacterium Paenibacillus sonchi: gene expression and physiological functions. Front Microbiol. 2020;11:588605. <https://doi.org/10.3389/fmicb.2020.588605>

Kobayashi T, Nozoye T, Nishizawa NK. Iron transport and its regulation in plants. Free Radical Bio Med. 2019;133:11–20. <https://doi.org/10.1016/j.freeradbiomed.2018.10.439>

Liang G. Iron uptake, signaling, and sensing in plants. Plant Communications. 2022;3(5):100349. https://doi.org/10.1016/j.xplc.2022.100349

Tsai HH, Schmidt W. One way. Or another? Iron uptake in plants. New Phytologist. 2017;214(2):500–505. <https://doi.org/10.1111/nph.14477>

Moreover, on page 9, line 165, there is a question about the term "multiple metabolisc pathways," with a suggestion that it might be a typographical error, possibly intended to be "metabolic.".

***Response:***

*Thanks, it’s our spelling mistakes. we changed "metabolic" instead of "metabolisc" in Line 170.*
