# Supplementary material for: Transcriptional alterations of peanut root during interaction with growth-promoting Tsukamurella tyrosinosolvens strain P9
Source: PLoS One. 2024 Feb 15;19(2):e0298303. doi: 10.1371/journal.pone.0298303 (PMC10868839; doi:10.1371/journal.pone.0298303)
Supplement: Attachment — Submitted filename: Response to Reviewers.doc [file pone.0298303.s014.doc]

Dear Editor,

Please find attached a revised version of our manuscript “Transcriptional alterations of peanut root during interaction with growth-promoting *Tsukamurella tyrosinosolvens* strain P9”.

Thank you for your kind letter regarding our manuscript, and the referees’ suggestions. We have revised and modified the MS in accordance with the every reviewers’ comments, and revisions in the text are shown using yellow highlight for additions, and strikethrough font for deletions. And we also responded point-by-point to reviewer comments as listed below. Accordingly, we added the cited references, and changed the serial number of the Reference because of some reference’s addition.

We hope that the revisions in the manuscript and our accompanying responses will be sufficient to make our manuscript suitable for publication in PLOS ONE. We shall look forward to hearing from you at your earliest convenience.

Yours sincerely,

First author, Xue Bai;

Corresponding author, Lizhen Han.

Corresponding author E-mail: [lzhan1@gzu.edu.cn;](mailto:lzhan1@gzu.edu.cn;) [hanlizhen11@163.com](mailto:hanlizhen11@163.com)

**Responses to the comments of Reviewer 1**

The modifications made to the manuscript, particularly in response to the previous comments have significantly improved the clarity and precision of the text. The provided link is now functional, addressing a previous issue. Overall, the study presents valuable insights; however, I have some specific comments and suggestions to enhance the clarity and precision of the manuscript.

1. Line 56-59: The statement "….to investigate their interaction" suggests that transcriptome analysis confirms the interaction, which may be misleading. Transcriptome analysis provides an overview resulting from the interaction, if any, but does not confirm it. I recommend modifying the sentence to accurately convey this point. Additionally, the remark, "...aimed to explore the molecular response mechanism," is somewhat ambiguous, as the study did not unveil a molecular mechanism per se. Rather, it focused on elucidating transcriptional alterations in peanut due to Tsukamurella tyrosinosolvens strain P9 inoculation. Therefore, I suggest refining the statements to align more closely with the study's actual findings.

***Response:***

*Thanks for your suggestion. We changed “to study the gene expression of peanut roots after inoculation with the growth-promoting P9 strain” instead of “to investigate their interaction”. And in order to make our studying aim clearly, we modified this sentence, that is, “our aim was to provide direct evidence for the molecular mechanism of strain P9 in promoting peanut growth, and to provide a theoretical basis for using Tsukamurella tyrosinosolvens strain P9 to the field level”.*

2. Line 162: Please consider removing the word "were" to improve the sentence structure..

***Response:***

*Thanks, we deleted this words.*

3. In my initial review, I mentioned the potential significance of the study's novelty. I would like to emphasize that if the authors can further highlight the significance of their study, which may not be adequately underscored in the current manuscript, it would substantially enhance the paper's potential for publication..

***Response:***

*It’s my negligence, your suggestion made our studying more meaniful. Thanks. We added some studying progress about this rare actinobacteria, Tsukamurella tyrosinosolvens in Line 49-53, and highlighted the novelty of this strain in Line 55.*

**Responses to the comments of Reviewer 2**

The manuscript has significantly improved as the authors have addressed the required revisions. The study was conducted on the molecular response mechanism of peanut seedlings to strain P9. They analyzed the transcript alterations to see the growth-promoting effects of strain P9 on peanuts. However, they could have enhanced the manuscript by incorporating a working model that elucidates the molecular mechanisms underlying the growth-promoting effects, providing a more comprehensive understanding of their claims.

***Response:***

*Thanks for your suggestion. In fact, the research on strain P9 has been ongoing. Since we isolated this strain of rare actinobacterium with strong phosphorus solubilization, Tsukamurella tyrosinosolvens P9, we found that this strain also has multiple growth-promoting properties such as secretion of IAA and siderophore, which can significantly promote the growth of peanut; while the underlying mechanism of its growth promotion is unclear, thus we tried to analyze it in a comprehensive way from different perspectives and aspects. It is well known that PGPR has a complex interaction with plants. In the previous study, we collected peanut root extraction (REs) and explored the effect of its addition on the transcriptome of P9 strain, especially on the expression of genes related to the growth-promoting function, and found that the peanut REs could significantly promote the growth and growth-promoting characteristics of P9 (Jiang et al. 2023). In this study, we established a "P9 strain-peanut co-culture system" and analyzed the transcriptome of peanut after inoculation with P9 strain, in order to explore how P9 strain promotes the growth of peanut from the perspective of gene expression. In this way, we can comprehensively assess the performance of P9 strain in the growth promotion function of peanut, so we believe that the paper is relatively comprehensive and complete.*

*Of course, your suggestion is very much appreciated, and we realise that if P9 and peanut in the co-culture system were studied simultaneously, the study would be more in-depth and more meaningful conclusions would be obtained. In fact, we are still conducting in-depth research on the growth-promoting mechanism of this strain; as P9 is a rare actinomycete, we are currently exploring and constructing a genetic transformation system of P9 strain to elucidate its growth-promoting properties on peanut growth; in addition, we have also found that P9 enhances drought resistance in peanut, and we are now studying using the co-culture system, hoping that we can more clearly analyze the mechanism of PGPR-plant interaction under adverse conditions.*
